# Optical gain reduction caused by nonrelevant subbands in narrow-period terahertz quantum cascade laser designs

**DOI:** 10.1038/s41598-022-25139-9

**Published:** 2022-12-23

**Authors:** Li Wang, Tsung-Tse Lin, Ke Wang, Hideki Hirayama

**Affiliations:** 1 RIKEN Center for Advanced Photonics, THz Quantum Device Team, 519-1399 Aramaki-aza Aoba, Aoba-ku, Sendai, 980-0845 Japan; 2 School of Electronics Science and Engineering, Nanjing University, 163 Xianlin Street, Qixia District, Nanjing, 210046 China

**Keywords:** Nanoscale devices, Nanoscale materials, Nanoscience and technology

## Abstract

The recent designs of terahertz quantum cascade lasers usually employ the short periodic length and also the tall barriers for high-temperature operation. In this work, the effect of high-energy lying non-relevant subbands is studied based on nonequilibrium Green’s function formalisms model, demonstrating those subbands are probable to play a minor role on the population inversion, but play a major role on the optical gain at high temperatures. The phenomenon can be ascribed to the appearance of leakages crossing neighboring periods via sequential resonant tunneling, and those leakages are inherently created by the specific features of the two-well configuration in this design that the phonon well should be wide enough for performing the phonon scattering to depopulate the lower-laser subband. The narrower periodic length design can strengthen this inter-period leakage. A parasitic absorption between the first high-lying nonrelevant subbands from two laser wells can closely overlap the gain shape and thus significantly reduce the peak gain.

## Introduction

Although the terahertz (THz) spectrum has been extensively applied in scientific and commercial fields^1,2^, the limited studies are conducted on because of the difficulty in generating THz waves using conventional semiconductor devices. Quantum cascade laser (QCL)^3^, a type of electrically pumped semiconductor laser based on repeating quantum heterostructures, is a promising candidate for the compact and high-power THz source. These devices are based on unipolar quantum confined carriers tunneling between discrete subbands. To date, THz-QCLs exhibit high emission output powers over 1 Watt in pulse operation^4^ and covers the laser frequency of 1 ~ 6 THz^2^. However, the main limitation is the operation still necessitates additional cryostat or thermo-electric cooling, thus preventing the THz widespread applications. Numerous studies have been conducted on revealing the background thermally degradations mechanisms in THz-QCLs, including the thermally activated longitudinal optical phonon (LO-phonon) non-radiations^5,6^, carriers leakage^7^, spectrum line broadening^8^, and thermal backfilling^9,10^. Various designs have been proposed to maximize the lasing temperature (T_*max*_), by suppressing those degradation processes (i.e., maintain the optical gain over the lasing threshold at high temperature)^11–15^. The design strategies can be summarized to promote the tunneling efficiency of each laser regions, (*a*), by employing the levels alignment resonances at the injector region^12,13^; (*b*), by using a diagonal intersubband radiation transition at the active region^12,16^; (*c*), by employing the direct LO-phonon resonance at the extractor region^13^. Meanwhile, the designs also intend to fulfill all those strategies simultaneously within a simplified quantum structure. It is because, for any given wavelength, resonator losses, and broadening lifetimes, the smaller number of relevant quantum subbands (that means a shorter periodic length) will directly contribute to the population inversion more. The historic high-T_*max*_ designs indeed follow a path to make the periodic length narrowing, i.e., a T_*max*_ of 140 K based on five subbands scheme^15^, a T_*max*_ of 199.5 K based on four subbands scheme^16^, a T_*max*_ above 200 K based on three subbands scheme^17,18^.

To ensure intersubband lasers electrically stable^3^, a QCL period requires an injector and minimum three relevant subbands which is basically based on at least two quantum wells. In designs with short period, a tall barrier is helpful to reduce fast leakage of electrons from relevant states directly into the continuum^6^ a 30% of AlAs% barrier within GaAs/AlGaAs quantum-well system provides a conduction band offset (CBO) of 300 meV, which can almost suppress leakages via up-scatterings into continuum. However, a tall barrier will confine more high-lying subbands (HLS) deeply. Here we define those confined high-lying subbands as “non-relevant” subbands. In a fact, in the early QCL design, those HLS are intentionally neglected for simplifying the model, but the two-well design emphasizes on the significant role of those HLS thanks to the specific configuration of short periodic quantum structure. In details, as each period only contains two quantum wells, among one should be as the phonon well which needs to be wide enough to keep an energy separation between its first excited state and ground state ≥ 36 meV^17–19^. By doing this, it can perform the efficient LO-phonon resonances to depopulate the lower-laser subband in a vertical transition way, thus maintaining high population inversion. This process also offers more robustness on the layer growth deviation and local doping than its ancestor design which employs resonant-tunneling for the depopulation, as the strict alignment condition needed for resonant-tunneling process can be easily broken^20^. However, this phonon well will naturally let downward its HLS in energy. Furthermore, when the lasing frequency is determined, this design has almost fixed periodic applied bias, that roughly equals to the depopulation energy plus one photon energy. If the periodic length become shorter, the stronger operating electric field will lower the down-stream HLS more due to the stark effect, which is more severe for the two-well design employing the depopulation energy larger than one LO-phonon.

In this study, the nonequilibrium Green’s function (NEGF) formalisms are used to investigate the effects of HLS especially at high temperatures from a viewpoint of inter-period interactions, and reveals that, (*a*), although the HLS can significantly redistribute the electrons among the relevant subbands, they indeed do not deteriorate the population inversion much; (*b*), but, the peak of optical gains are obviously reduced and the gain shapes are severely deformed, that betray the trend of population inversion, which can be ascribed to the specific parasitic absorption overlapping the gain caused by the HLS. Therefore, the HLS are probable to play dichotomous roles on population inversion and the gain.

## Results and discussion

**Figure 1:**
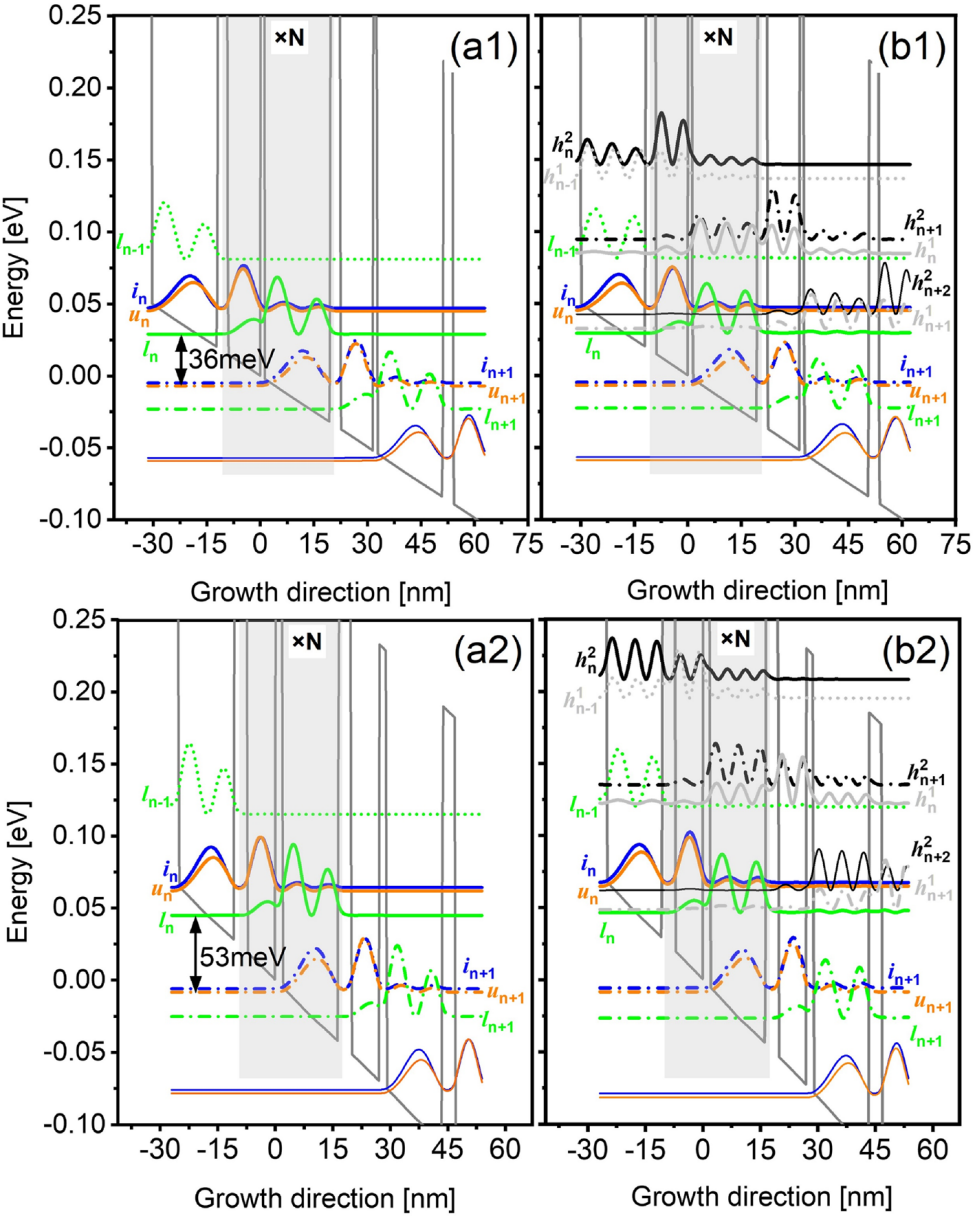
GaAs/Al_0.3_Ga_0.7_As conduction band diagram of the short-period resonant-phonon design at an operating bias within three neighboring periods (*n* − 1, *n*, and *n* + 1). Two different designs are shown with the depopulation energy of 36 meV and 53 meV, respectively. The widest wells in both designs are locally doped with a 2D electron density of 4.6 × 10^10^ cm^−2^. The wavefunctions of confined subbands in quantum heterostructures are displayed, such that (**a1**, **a2**) only contains three relevant subbands in the model, the injector subband *i*, the upper-laser subband *u* and lower-laser subband *l*, as [*i*, *u*, *l*] (**b1**, **b2**) beside the relevant subbands, also the HLS are involved in the model, as [*i*, *u*, *l*, *h*^1^, *h*^2^].

Figure 1 displays the two-well resonant-phonon THz-QCLs design using the tall barriers (30% of AlAs% in AlGaAs), here two different depopulation energy are used, i.e., 36 meV-design (Fig. 1a1,b1) and 53 meV-design (Fig. 1a2,b2). Three neighboring periods are shown (labeled as *n* − 1, *n*, *n* + 1). The electrons are firstly injected from *i*njector subband *i* into the *u*pper-laser subband *u* by resonant-tunneling with a resonance anticrossing energy of 2.5 meV, and the population is intentionally inverted between subbands *u* and the *l*ower-laser subband *l*. The lasing behavior follows a diagonal transition manner, and the diagonality of this radiation transition is quantified with an oscillator strength of 0.28. The depopulation of *l* is achieved following the direct-phonon resonance in the same well, merely by engineering the eigenenergy separation of *l* and *i'* (the next injector subband) at ≥ 36 meV. The electrons then will repeat the previous transport steps in the down-stream periods. Therefore, in each period, the whole quantum transport passing the relevant subbands proceeds following *i → u → l → i'*. To study the effects of HLS, the axial cut-off energy is controlled to obtain a small scale that contains only three relevant subbands [*i*, *u*, *l*] (ideal but not real for QCLs), and also a full scale including more HLS (real for QCLs), noted here, the most correlated HLS in this two-well resonant-phonon design are *h*^1^ and *h*^2^, hereafter, we mainly focus on those two HLS in this work to save the computing cost.

**Figure 2:**
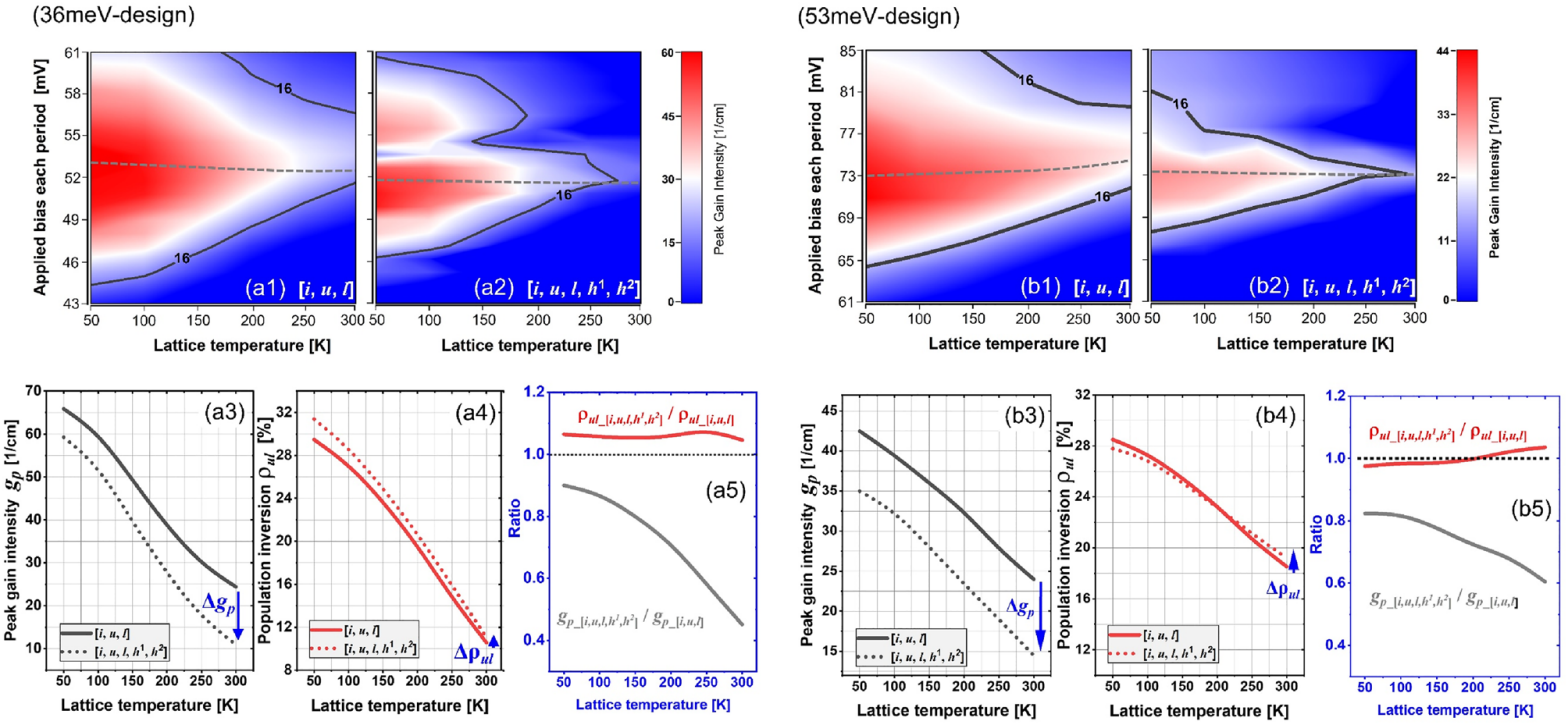
Peak gain mappings resolved by the lattice temperature and period applied bias from both the designs, including the mappings based on only relevant subbands, [*i*, *u*, *l*] **(a1, b1)**, and the mappings based on subbands including HLS, [*i*, *u*, *l*, *h*^1^, *h*^2^] (**a2**, **b2**). The black solid line boundary indicates the cavity threshold at 16 cm^−1^ for lasing. The gray dash line represents the bias at an operating condition. **(a3, a4; b3, b4)** Comparations of the peak gain *g*_*p*_ (extracted from the gray dash line in the mappings (**a1**, **a2**, **b1**, **b2**) and also the population inversion ρ_*ul*_, with/without the HLS. (**a5**, **b5**) HLS-induced changing magnitude for both the peak gain and population inversion. Here, ∆ρ_*ul*_ (+) denotes an increasing changing trend in the population inversion, ∆*g*_*p*_ (−) denotes a decreasing changing trend in the peak gain.

Figure 2 displays the optical gain mappings resolved by the bias applied on each period (*y*-scale) and the lattice temperatures (*x*-scale) for both scales with/without the HLS. The solid black line indicates the laser cavity threshold where the gain more than this value demonstrates lasing potentials. Here, a cavity loss (including both the waveguide and mirror losses) is assumed at 16 cm^−1^ based on a metal–metal Fabry–Perot (F–P) cavity structure. The gray dash line indicates the operating bias condition at different temperatures. For 36 meV-design, in a case of small scale [*i*, *u*, *l*] (Fig. 2a1), as temperature increasing, the gain surrounded by threshold boundary shrinks, i.e., at 50 K, the *y*-bias range is 21 mV, and at 300 K, this range become considerably small to 6 mV. However, the surrounded gain area is almost symmetric, and remains even till room temperature. After the HLS are included as a scale [*i*, *u*, *l*, *h*^1^, *h*^2^] (Fig. 2a2), it is clear that the surrounded gain becomes asymmetric, and shrinks more as compared with the [*i*, *u*, *l*] scale case, i.e., a *y*-bias range of 14 mV at 50 K, and become 0 mV at 270 K. For 53 meV-design, in [*i*, *u*, *l*] scale (Fig. 2b1), the gain surrounding *y*-bias ranges are 26 mV at 50 K and 8 mV at 300 K, slightly larger than that in 36 meV-design. In [*i*, *u*, *l*, *h*^1^, *h*^2^] scale (Fig. 2b2), the HLS leads to the *y*-bias range shrinking to 13 mV at 50 K, but this range keeps more than 0 till 290 K. Figure 2 also show the peak gain *g*_*p*_ and also the corresponding population inversion ρ_*ul*_ (= ρ_*u*_ − ρ_*l*_) under the operating bias condition. It is true that the 53 meV-design indeed demonstrates larger *g*_*p*_ and ρ_*ul*_ at high temperatures than that of 36 meV-design for both [*i*, *u*, *l*] and [*i*, *u*, *l*, *h*^1^, *h*^2^] scales. As a guide of eyes, the ratio of *g*_*p*_, ρ_*ul*_ of both scales are also displayed. Notably, the inclusion of HLS actual plays a minor role on the changes of the ρ_*ul*_ at high temperatures, i.e., the ratio of ρ_*ul_*[*i*, *u*, *l*, *h*1, *h*2]_/ρ_*ul_*[*i*, *u*, *l*]_ are 1.04 and 1.03 at 300 K for 36 meV-design and 53 meV-design, respectively. However, significant difference can be observed on the *g*_*p*_, i.e., the ratio of *g*_*p_*[*i*, *u*, *l*, *h*1, *h*2]_/*g*_*p_*[*i*, *u*, *l*]_ are 0.45 and 0.6 at 300 K for 36 meV-design and 53 meV-design, respectively, indicating the HLS can cause a strong reduction on the peak of gain. It therefore needs to answer, at high temperatures, when the HLS are included, *question-*1: does the almost unchanged population inversion mean no redistribution of population among the subbands by HLS; *question-*2: following the gain equation *g*_*p*_ ~ *d*_*ul*_^2^·ρ_*ul*_/*Γ*_*ul*_, the *g*_*p*_ should also be changed little due to the unchanged ρ_*ul*_ (the dipole elements *d*_*ul*_ and also the transition linewidth *Γ*_*ul*_) are almost constant in cases with/without the HLS), why the *g*_*p*_ is reduced so much.

**Figure 3:**
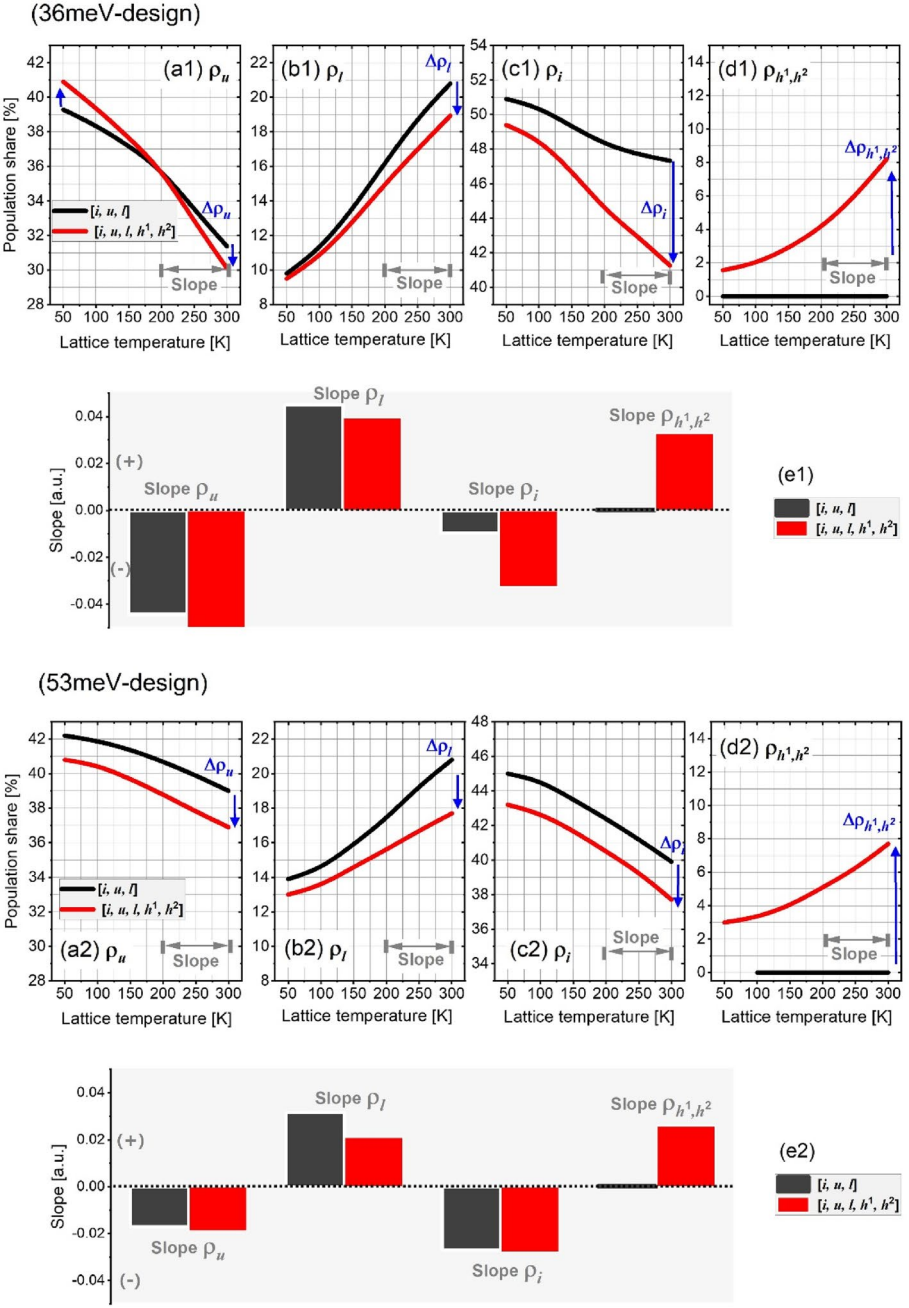
Population fractions at each subbands as a function of temperatures in both designs (**a1**–**d1**, 36 meV-design; **a2**–**d2**, 53 meV-design). Here, ρ_*u*_, ρ_*l*_, and ρ_*i*_ denote the fraction at upper-laser, lower-laser, and injector subbands, ρ_*h*1,*h*2_ is a sum of fractions at both the HLS, *h*^1^ and *h*^2^. The black/red lines represent the small scale [*i*, *u*, *l*] and the full scale [*i*, *u*, *l*, *h*^1^, *h*^2^], respectively. (**e1**, **e2**) The changing slope of population fractions at high temperatures above 200 K. The black rectangles indicate the small scale [*i*, *u*, *l*] and the red rectangles is for a full scale [*i*, *u*, *l*, *h*^1^, *h*^2^]. The plus/minus slopes denote the increasing/decreasing changing trend at high temperatures above 200 K, respectively.

For *question-1*, we first clarify the electrons distribution between only three relevant subbands (a small scale [*i*, *u*, *l*]) depending on the temperatures. By doing this, the observations can then support the analysis of the redistribution in the compliant case where the HLS are considered. As shown by black solid lines for both designs in Figure 3, no matter the temperature low or high, the subband *i* always possess most population (with a fraction ~ 50%) thanks to the reversibility of the resonance alignment and the relative thick injector barrier. The population fractions show different trends as temperature increasing, that is, both the fractions of subbands *u* and *i* (ρ_*u*_, ρ_*i*_) decrease, but the fraction of subband *l* (ρ_*l*_) increases. The summed decreasing magnitude of both the ρ_*u*_ and ρ_*i*_ is almost equal to that of ρ_*l*_, that indicates the electrons seems to redistribute from subbands *u* and *i* into *l* finally as the temperature increases. By comparing the net changing of the ρ_*l*_ when the temperature is increased from 50 to 300 K, the 53 meV-design is smaller than 36 meV-design, i.e., Δρ_*l*_ in 36 meV- and 53 meV-design are 11.8% and 7.5% fractions, respectively.

The more changing Δρ_*l*_ in 36 meV-design is actual ascribed to the thermal backfilling (*i*_*n*_/*u*_*n*_ → *l*_*n*-1_), whereas such a thermal backfilling is partially suppressed by the much larger depopulation energy.

After the HLS are included, the corresponding population fractions at each subband are shown in Fig. 3 by the red solid lines. The changing fraction (Δρ) in this scale as compared with the small scale [*i*, *u*, *l*] is marked by blue arrows. Firstly, no matter the temperatures are, the ρ_*l*_ decreases that proves the role of HLS can be as an additional depopulation channel for subband *l*, and this channel seems to more effective for 53 meV-design (Δρ_*l*_, arrow down in Fig. 3b1,b2). The effects of HLS on the ρ_*u*_ are different in both designs, for 36 meV-design, the ρ_*u*_ increases at a temperature below 170 K (Δρ_*u*_, arrow up) and then decreases when the temperature is above 170 K (Δρ_*u*_, arrow down) (36 meV-design Fig. 3a1). It is because, at a low temperature, the electrons leaking into HLS will partially relax down to the subband *u* by increasing it, but as the temperature increasing, the thermally up-scattering from subband *u* directly into the HLS can lead to a net decrease on the ρ_*u*_. For 53 meV-design, the HLS results in a continuous decrease on the ρ_*u*_ at both low and high temperatures (Δρ_*u*_, arrow down in Fig. 3a2). This different trend of ρ_*u*_ in both design is mainly because an additional leakage channel activated via u_n_ → *h*_*n*+1_^2^ for 53 meV-design is more effective than that of 36 meV-design, as shown in Fig. 1b1 and b2. For population shared by HLS, a fraction of *h*^1^ and *h*^2^ can be as high as 8% in both designs at 300 K.

**Figure 4:**
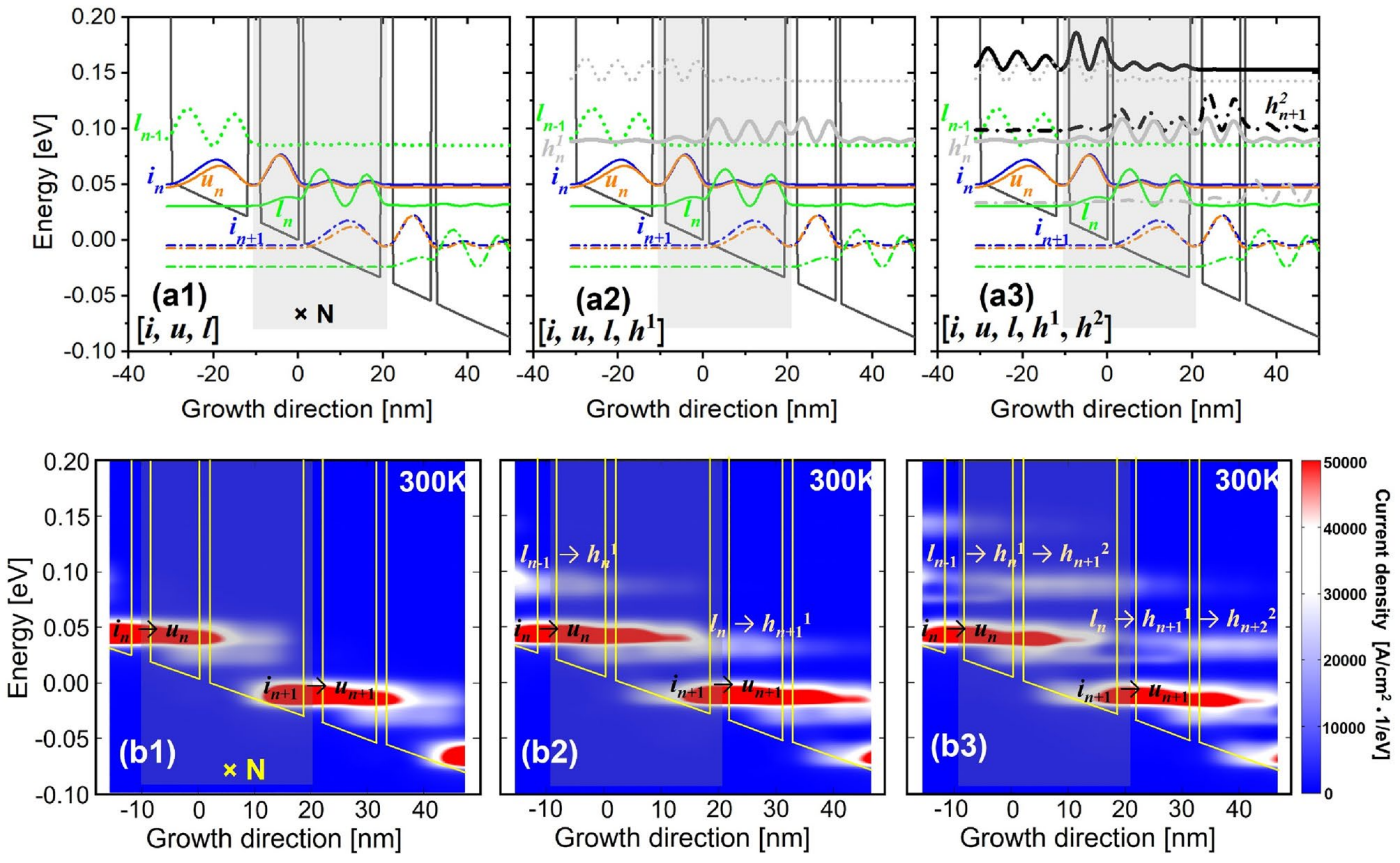
Current density mappings resolved by position and energy at high temperature of 300 K as the HLS is included one by one. (**a1**, **b1**) for [*i*, *u*, *l*], (**a2**, **b2**) for [*i*, *u*, *l*, *h*^1^], (**a3**, **b3**) for [*i*, *u*, *l*, *h*^1^, *h*^2^].

Figure 3-e displays the changing slope at high temperatures > 200 K. It is clear that, in 36 meV-design, the slopes of ρ_*u*_ and ρ_*l*_ are almost not changed when including or excluding the HLS (i.e., the similar height of the black and red bars in Fig. 3e1). But the slopes of ρ_*i*_ and ρ_*h*1*,h*2_ are very sensitive to the HLS where the red bar height is much taller than the black bar. As stated above, the ρ_*h*1*,h*2_ actual represents a leakage from the relevant subbands. It is immediate to reach an impression that, at high temperatures, the emerging of HLS mainly redistributes the population between the subband *i* and HLS *h*^1^/*h*^2^. To verify this speculation, by including the HLS *h*^1^ and *h*^2^ one by one (Fig. 4a1–a3), the resonance parasitic channels can be formed crossing three neighboring periods (*l*_*n*−1_* → h*_*n*_^1^* → h*_*n*+1_^2^) as shown in the 300 K position-resolved current mappings (horizontal bar in Fig. 4b1–b3). The thermal backfilling process is dominant via a path *i*_*n*_* → l*_*n−*1_ at high temperatures in 36 meV-design. Therefore, this parasitic channel firstly follows a “vertical” thermal backfilling process and subsequently follows the “horizontal” path, as *i*_*n*_ → *l*_*n−*1_ → *h*_*n*_^1^ → *h*_*n*+1_^2^. For 53 meV-design, the slope of ρ_*u*_ is smaller than that of 36 meV-design, and almost constant after including the HLS. There is a similar trend for the slopes of ρ_*i*_ (almost constant). Instead, the ρ_*l*_ and ρ_*h*1*,h*2_ suffer from an obvious changing slope at high temperatures thanks to the HLS emerging. The reason is that the thermal backfilling in 53 meV-design is already well suppressed by a large depopulation energy, but it partially sacrifices the depopulation efficiency of subband *l*. Therefore, at high temperature, more populations intend to reside at subband *l*. As the periodic length of 53 meV-design is narrower, the parasitic coupling between the subband *l*_*n*_ with the downstream HLS *h*_*n*+1_^1^ become stronger (Fig. 1d), it can be more effective to extract the residual populations at subband *l* via long-range resonant tunneling. The population of *h*^1^ and *h*^2^ are mainly from those tunneling.

**Figure 5:**
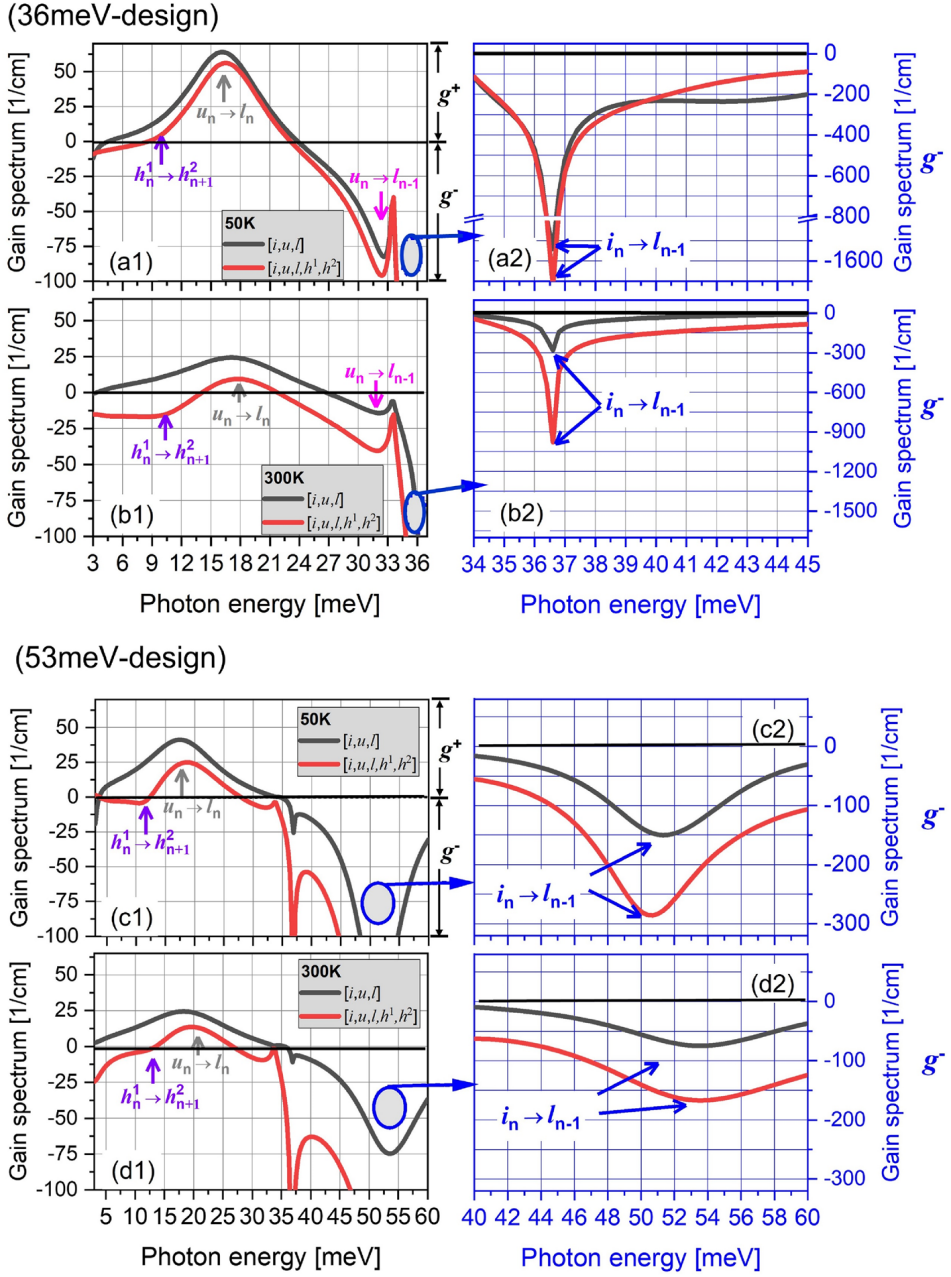
Gain spectrums in cases of a small scale [*i*, *u*, *l*] (black curves) and a full scale [*i*, *u*, *l*, *h*^1^, *h*^2^] (red curves) in both designs at 50 K (**a1**, **c1**) and 300 K (**b1**, **d1**). The exact transitions between subbands pairs are labeled by different color arrows in the spectrums. The gain is marked as ***g***^**+**^, and the absorption is marked as ***g***^***−***^. The exact absorption area near the depopulation energy is enlarged in plots (**a2**, **b2**).

For *question-2* regarding the inconsistency between population inversion and gain (as firstly shown in Fig. 2a5,b5), the gain spectrums of both the designs are shown with/without the HLS at both 50 K and 300 K (Fig. 5). It can be seen clearly the very strong absorptions at the photon energy of 36 meV/53 meV in 36 meV/53 meV-designs (negative gain area, *g*^*-*^), which represents the LO-phonon resonance absorption from *i*_*n*_ to *l*_*n-*1_ (Fig. 5a2–d2, enlarged plots). The peak at 18 meV is the optical gain thanks to the designed radiation transition from *u*_*n*_ to *l*_*n*_. Besides those pairs of subbands transition, there also emerges a parasitic absorption between the HLS *h*_*n*_^1^ and *h*_*n*+1_^2^, and this absorption has a peak position near 11 meV overlapping the gain peak at 18 meV severely. Here, we focus on each peak position to obtain more evidence. In details, (*a*), *for peak positions of 36 meV/53 meV-absorption* (*i*_*n*_ → *l*_*n*−1_): the inclusion of HLS actual results in stronger its absorption peaks. This enhancement is more obvious at high temperature than at low temperature with an enhanced magnitude, i.e., in 36 meV-design, 1.17 times at 50 K (*g*^(−)^__[*i*, *u*, *l*, *h*1, *h*2]_ = − 1600 cm^−1^, *g*^(−)^__[*i*, *u*, *l*]_ = − 1370 cm^−1^) and 3.6 times at 300 K (*g*^(−)^__[*i*, *u*, *l*, *h*1, *h*2]_ = − 800 cm^−1^, *g*^(−)^__[*i*, *u*, *l*]_ = − 220 cm^−1^); In 53 meV-design, 1.9 times at 50 K (*g*^(−)^__[*i*, *u*, *l*, *h*1, *h*2]_ = − 285 cm^−1^, *g*^(−)^__[*i*, *u*, *l*]_ = − 150 cm^−1^) and 2.2 times at 300 K (*g*^(−)^__[*i*, *u*, *l*, *h*1, *h*2]_ = − 165 cm^−1^, *g*^(−)^__[*i*, *u*, *l*]_ = − 75 cm^−1^); (*b*), *for the peak position of 11 meV-absorption* (*h*_*n*_^1^ → *h*_*n*+1_^2^): this absorption is very near the gain and forms an overlap. For 36 meV-design, at a low temperature, this absorption already emerges and causes slight deviation of spectrum shapes in [*i*, *u*, *l*] and [*i*, *u*, *l*, *h*^1^, *h*^2^] (at a photon energy range between 9 and 12 meV in Fig. 5a). As compared, in 53 meV-design, this absorption severely deforms the gain spectrum shape at 50 K thanks to the much stronger coupling of HLS *h*^1^ and *h*^2^ than that in 36 meV-design. Noted that, at high temperature of 300 K, this absorption can lead to serious deformation on the gain shape in both designs, showing strong concave curve in the gain spectrums. Therefore, the inconsistency of population inversion and gain seems to be ascribed to this parasitic absorption.

In such a short-period design, the quantum structure configurations inherently hold the limitations on those HLS, where the inter-period parasitic channels *l → h*^1^* → h*^2^ are easily activated and enhanced as temperature increasing. For much narrower design, i.e., 53 meV-design, the upper-laser subband also can participate in these channels (*l → h*^1^; *u → h*^2^). In details, (*a*), the direct-phonon depopulation process relies on the engineering of ground and 1st excited subband in phonon well, thus this well will have a large width. The HLS from this phonon well will be downward in energy. Meanwhile, the electric filed become much stronger as the periodic length is narrower. As a result, the downstream HLS can be further lower thanks to the stark effect; (*b*), the direct-phonon process suffers more from thermally backfilling at high temperatures as compared with the resonant-phonon scheme; (*c*), in addition, at high temperatures, the degraded resonant tunneling due to thermally level broadening can result in doublet shortcomings, one is to make the injection efficiency lower that leads to more populations residual at injector subband *i*, second is to suffer from thermally backfilling more serious thanks to this populations at subband *i*, especially in a case of relative small depopulation energy design, i.e., 36 meV-design in this work. Based on this, although the much larger depopulation energy design can convincingly suppress the thermal backfilling, the further strategies in this short-period design are needed to relax this parasitic absorption for further improved T_*max*_, one example as shown in Ref.^21^, by using a split-well structure to push up all the nonrelevant subbands.

## Conclusion

In summary, two-well resonant-phonon designs with different depopulation energy is studied based on a NEGF model, it shows that the HLS is probable to play different roles on population inversion and optical gain, that minor effecting on reducing the former one, but significantly reducing the latter one. Those dichotomous roles are attributed to the appearance of specific parasitic absorption overlapping the gain. Such an absorption is original from the first high lying nonrelevant subbands from both the upper and lower wells, where the residual population on them are leaked from the upstream laser subbands via resonant tunneling.

## Method

The fundamental tool in the THz-QCLs designs and analysis relies on a numerical package to calculate subband wavefunctions and energies. Because the parasitic channels discussed in this work are sensitive to the detuning energy of coupled subbands, it requires to estimate the subband energy position more precisely. Here, two factor effecting the detuning energy between subbands are considered, (*a*), In THz-QCLs, the quantum structure contains the layers with thickness of only several nanometers, the nonparabolicity can largely affect on the confined subband energy^22^, especially on the HLS as it is lifted further away from the bottom of the conduction band. The high-electric filed operation of THz-QCLs can make this issue worse. Here, the band structures are based on three-band Hamiltonian that accounts for the conduction (*c*), light-hole (*lh*), and split-off (*so*) bands, as follows:

1 $$H=\left(\begin{array}{ccc}{E}_{c}\left(z\right)+S(z)\frac{{\hslash }^{2}{k}_{z}^{2}}{2{m}_{0}}& i\sqrt{\frac{2}{3}} P(z){k}_{z}& -i\sqrt{\frac{1}{3}} P(z){k}_{z}\\ -i\sqrt{\frac{2}{3}} P(z){k}_{z}& {E}_{lh}\left(z\right)+(1+L(z))\frac{{\hslash }^{2}{k}_{z}^{2}}{2{m}_{0}}& 0\\ i\sqrt{\frac{1}{3}} P(z){k}_{z}& 0& {E}_{so}\left(z\right)+(1+L(z))\frac{{\hslash }^{2}{k}_{z}^{2}}{2{m}_{0}}(z)\end{array}\right)$$

where *P* is the interband momentum matrix element related to the Kane energy *E*_*p*_ through:$P\left(z\right)=\sqrt{\frac{{m}_{0}{E}_{p}(z)}{2}}$. By comparing the three-band and one-band models for calculating the HLS energy, it finds an approximate 2 meV difference; (*b*), the alignment of subbands is also very sensitive to the conduction band offset (CBO) values, especially in short-period design with tall barriers (here, AlAs% of 30% in AlGaAs barrier). We follow the latest calibration of CBO in tall barriers based on a machine-learning method reported in Ref.^18^.

The exact numbers of subbands participating in the transports are manually controlled by the axial cut-off energy. These subbands are transformed into localized basis modes (reduced real space basis) and used in the NEGF algorithm. The subband energy broadening can play significant roles for estimating the tunneling current (by increasing the dephasing) and the optical gain (by widening the radiation linewidth), especially for THz-QCL studied in this work, because this subband energy broadening (~ 10 meV) is already similar as the photon energy (15 meV). In THz-QCLs, this broadening originates from multiple scattering couplings. Here, the self-energy terms are used for all scatterings, including the optical phonons, acoustic phonons, charged-impurities, interface roughness, alloy disorder, and electron–electron interactions^23–25^. The critical part of the model is a self-consistent NEGF solver that starts from an initial guess of the Green’s functions, the self-energies are then presented roughly, the Green’s functions are again calculated iteratively. Simultaneously, the mean-field electrostatic potential is calculated self-consistently (Poisson’s equation). Such iterations are performed until convergence is reached. The current density as well as the carrier density distribution is finally displayed. The optical gain or absorption in pairs of intersubband transitions follows the semiclassical way, *i*.*e*., at the photon energy of $\hslash \omega$ according to the following expression:

2 $$g\left(\hslash \omega \right)=\sum_{i\ne j}{n}_{3D}\left({\uprho }_{i}-{\uprho }_{j}\right){d}_{ij}^{2}\frac{{\Gamma }_{ij}}{{(\hslash \omega -{E}_{ij})}^{2}+\frac{{\Gamma }_{ij}^{2}}{4}}\frac{{e}^{2}{E}_{ij}}{ c\hslash {\epsilon }_{0}\sqrt{{\epsilon }_{r}}}$$

where ρ_*i*_ is the normalized population fraction of subband *i*, and *n*_3D_ is the averaged 3D electron density in each period. *e* represents the electron charge. *d*_*ij*_ is the dipole of the intersubband transition. *E*_*ij*_ is the transition energy separation between subbands *i* and *j* that equals *E*_*i*_ – *E*_*j*_. Here, *Γ*_*ij*_ is the transition linewidth (full half at half maximum), which closely depends on subband energy broadenings. Furthermore, ${\epsilon }_{r},{\epsilon }_{0}$ is the relative and vacuum permittivity respectively.

## Data Availability

The datasets used and/or analyzed during the current study available from the corresponding author on reasonable request.
